# Allergen Content of Inactive Ingredients in Best‐Selling Sunscreens: A Comparison of Key Product Features

**DOI:** 10.1111/cod.70141

**Published:** 2026-04-12

**Authors:** Emily J. Levin, Christopher Chung, Katia Hermes, Henry W. Lim, Natalie H. Matthews

**Affiliations:** ^1^ School of Medicine University of Pittsburgh Pittsburgh Pennsylvania USA; ^2^ Department of Dermatology Henry Ford Health Detroit Michigan USA; ^3^ College of Human Medicine Michigan State University East Lansing Michigan USA; ^4^ Dean's Office, Michigan State University College of Human Medicine East Lansing Michigan USA; ^5^ Department of Medicine, Michigan State University College of Human Medicine East Lansing Michigan USA

**Keywords:** allergens, best‐selling sunscreens, contact allergy, contact dermatitis, inactive ingredients, NAC‐80, patch testing, sunscreen, sunscreen vehicle, UV filters

## Abstract

**Background:**

Irritant and allergic contact dermatitis (ACD) caused by sunscreens may affect patient adherence and photoprotection.

**Objectives:**

We aimed to compare the presence of *inactive* allergens from the North American 80 Comprehensive Series (NAC‐80) across top‐selling sunscreens.

**Methods:**

We conducted a cross‐sectional analysis of inactive ingredient lists from all reported best‐selling sunscreens from the three largest American online retailers: Amazon.com, Target.com and Walmart.com. Using a custom text‐matching algorithm and manual review, we identified NAC‐80 allergens and quantified allergen counts per product. We compared inactive allergen load in tinted versus non‐tinted, organic versus inorganic ultraviolet (UV) filters, vehicle and various marketed features in sunscreens.

**Results:**

We reviewed 176 products. Vitamin E was the most common allergen, followed by acrylate‐containing copolymers/crosspolymers, fragrance, and parabens. Organic, spray and sport sunscreens had significantly more allergens than inorganic, stick and non‐sport sunscreens, respectively. Tinted and face sunscreens had significantly fewer allergens than non‐tinted and body sunscreens.

**Conclusions:**

Allergen content in inactive ingredients varies among best‐selling sunscreens, potentially affecting their safety and tolerability. Dermatologists should consider the allergenic potential of not only active but also inactive ingredients of sunscreens when counselling patients.

## Graphical abstract

Allergenic risk of sunscreen active ingredients is well known, but that of inactive ingredients remains understudied. We identified and quantified allergens found in inactive ingredients of best‐selling sunscreens and developed a free tool (www.NAC80.com) that helps dermatologists recognise higher‐risk products and counsel patients prone to allergic or irritant reactions.

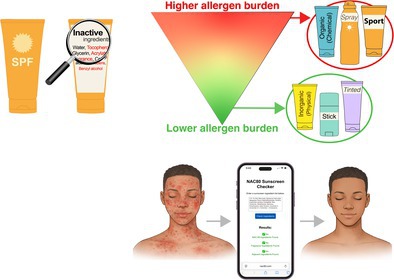

## 1 Introduction

Dermatologists frequently recommend daily sunscreen use to protect against photoaging and reduce skin cancer risk [1, 2, 3]. Allergic contact dermatitis (ACD) is a delayed type IV hypersensitivity reaction triggered by cutaneous exposure to sensitising allergens and affects approximately 15%–20% of the general population [4]. Sunscreen presents a unique risk in this context. It is often applied daily, over large surface areas, and sometimes multiple times per day. It also tends to be worn under occlusive layers like makeup or clothing, increasing dose and penetration. These factors may raise the likelihood of sensitization, especially when the product contains multiple known allergens.

It has long been recognised that UV‐filters such as oxybenzone, octinoxate and octocrylene can cause ACD [5, 6]. The inactive ingredient profile of sunscreens and potential propensity to cause an ACD is less understood. The inactive ingredients of sunscreen are added for purposes such as emulsion, preservation, fragrance or cosmetic desirability. These components may increase the risk of sensitization, particularly fragrances and preservatives, which are among the most common ACD triggers.

Patients with a history of ACD or sensitive skin may be discouraged from use if their sunscreen triggers rash. We seek to identify, quantify and categorise any potential allergens in inactive sunscreen ingredients.

## 2 Methods

### 2.1 Data Collection and Preprocessing

On May 4 and 5, 2024, all listed best‐selling sunscreens from the three largest U.S. online retailers (Amazon.com, Walmart.com and Target.com) were selected for review. Data on all listed ingredients (including inactive ingredients and UV filter), product vehicle (spray, lotion, stick) and the presence of the marketing keywords in product descriptions ‘tinted’, ‘sport’, ‘baby’ (or ‘child’), ‘face’ and ‘body’, were collected for 176 sunscreens.

We cross‐referenced inactive ingredients against the North American 80 Comprehensive Series (Chemotechnique Diagnostics, NAC‐80 Series) and identified and quantified listed allergens using an in‐house text‐comparison algorithm and manual review. The NAC‐80 panel was designed to identify the most common contact allergens in North America. Allergen classification and grouping followed clinical patch testing standards; details on the text‐matching algorithm and grouping logic (e.g., parabens, acrylates, tocopherol derivatives) are provided in the Supporting Information. For acrylates, grouping referred to acrylate‐containing copolymers and crosspolymers listed in ingredient lists rather than acrylate monomers.

### 2.2 Statistical Analysis

All statistical analyses were performed in R (4.2.2). Descriptive statistics were calculated for the number of NAC‐80 allergens found across all sunscreens. We calculated the average number of NAC‐80 allergens for each comparison category: UV filter (organic vs. combination vs. inorganic; refer to Supporting Information and Table S1 for categorization), vehicle (spray vs. lotion vs. stick) and marketing label (tinted vs. non‐tinted, sport vs. non‐sport, baby vs. adult, face vs. body). Averages were displayed in grouped barplots with standard error of the mean (SEM). Welch's *t*‐tests were used for binary comparisons, and one‐way ANOVA with Tukey's HSD for multi‐group comparisons. To visualise allergen distribution, we generated histograms for each sub‐category.

## 3 Results

**FIGURE 1:**
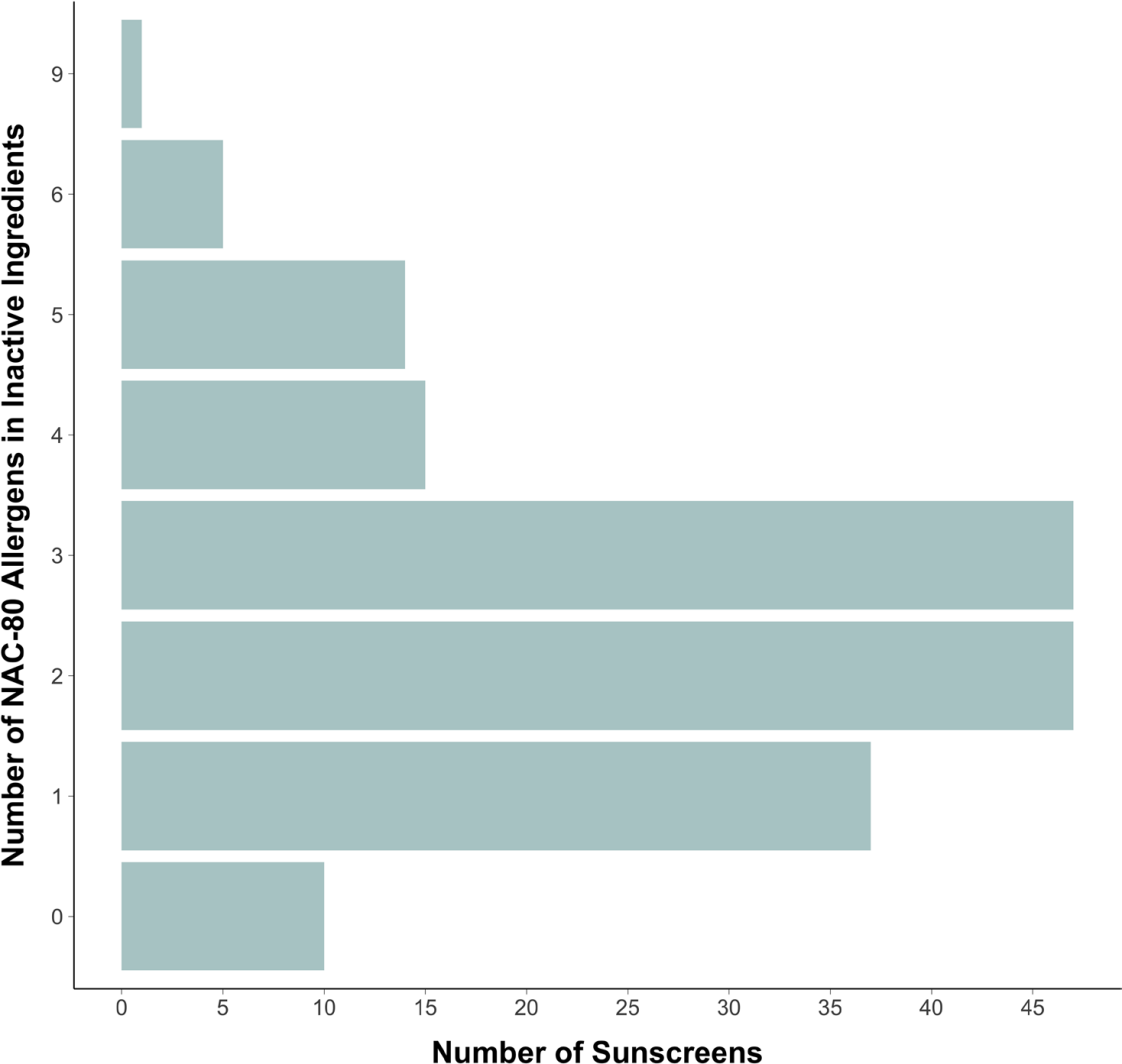
The number of NAC‐80 allergens in inactive ingredients of sunscreens. The *Y*‐axis is the number of allergens from inactive ingredients (e.g., 0 means sunscreens have 0 NAC allergens, 5 means sunscreens have 5 NAC allergens), and the *X*‐axis is how many sunscreens (out of 176 total sunscreens) contain a given number of allergens (e.g., ~47 sunscreens contain 3 allergens).

Across 176 best‐selling sunscreens, the mean number of NAC‐80 allergens among listed inactive sunscreen ingredients was 2.51 (SD = 1.5), with a median of 2 and a mode of 2. The full range was 0–9 allergens. Only 10 products (5.6%) contained zero allergens (Figure 1). Figure 1 displays the frequency of allergen counts in inactive ingredients across all products.

**FIGURE 2:**
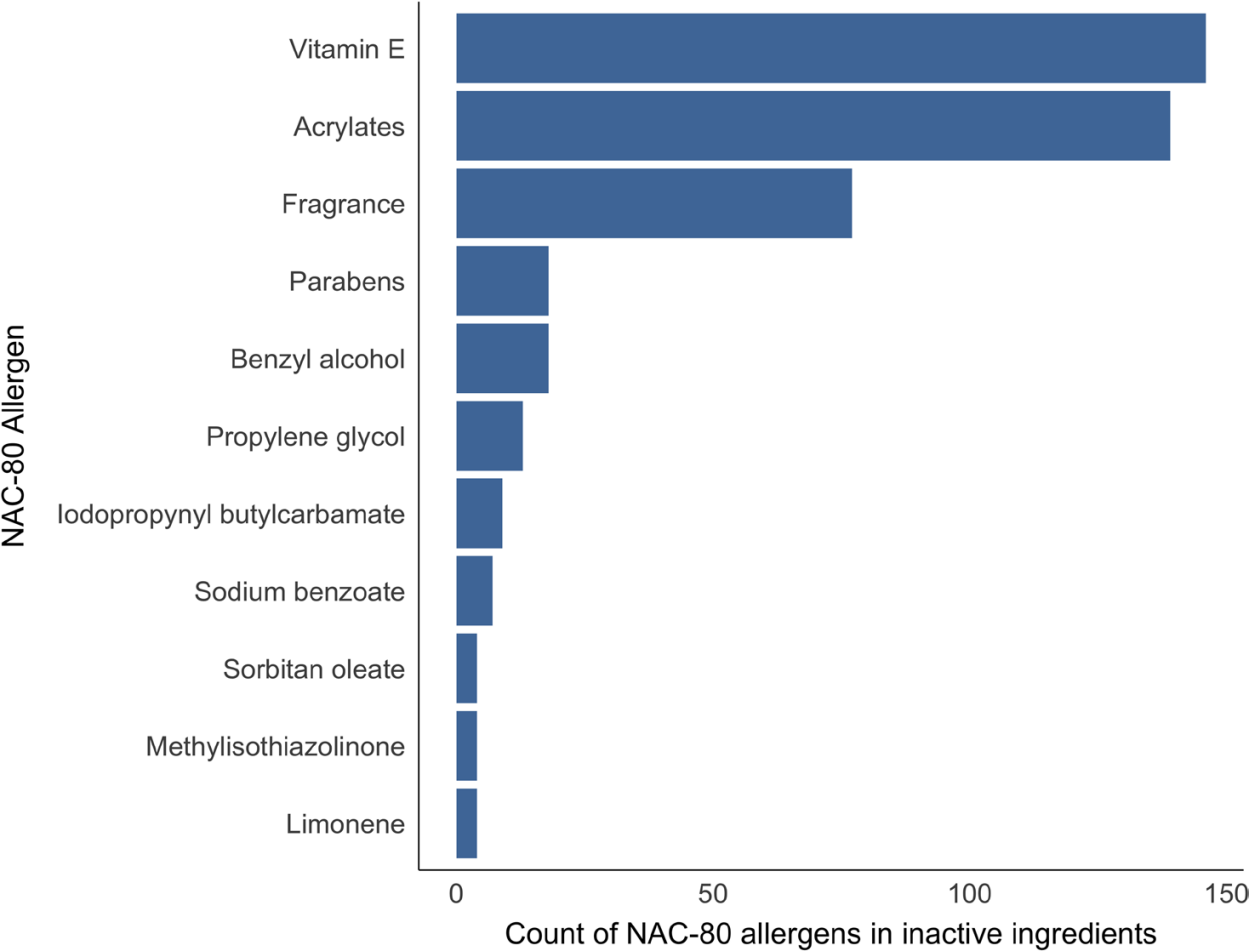
Frequency of top eleven most common NAC‐80 allergens in inactive ingredients of best‐selling sunscreens. Allergens are grouped by chemical class for clarity. Categories such as ‘Vitamin E’ include both tocopherol and tocopheryl acetate; “Acrylate polymers” includes all acrylate‐ and methacrylate‐containing copolymers and crosspolymers. Parabens include all paraben‐containing compounds. Eleven allergen categories are shown due to ties.

We identified 49 unique allergens within the inactive ingredient lists of best‐selling sunscreens (listed in Table S4). The most common unique allergen was fragrance (*n* = 77), followed by tocopherol (*n* = 74) and tocopheryl acetate (*n* = 72). When grouped by chemical class (e.g., combining tocopherol and tocopheryl acetate into “Vitamin E” and combining all unique acrylate‐containing copolymers/crosspolymers into one “acrylate polymers” category), the most common allergen was Vitamin E (tocopherol + tocopheryl acetate) (*n* = 146), acrylate polymers (*n* = 139), fragrance (*n* = 77), parabens (*n* = 18) and benzyl alcohol (*n* = 18). The top allergens by overall frequency are shown in Figure 2 (11 categories shown due to ties). See Figure S1 for list of 19 allergens.

**FIGURE 3:**
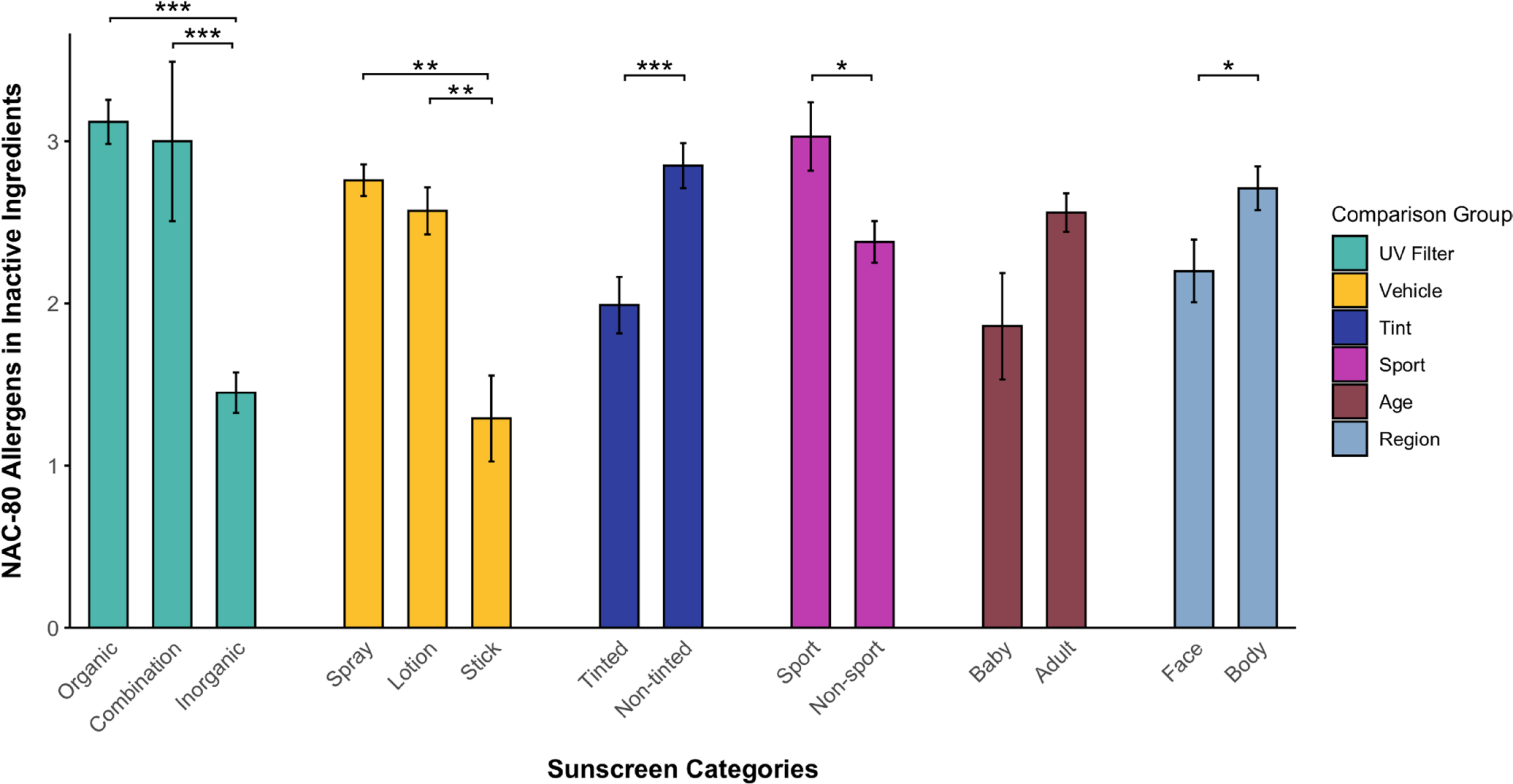
Number of NAC‐80 allergens in inactive ingredients of best‐selling sunscreens by UV filter, product vehicle, tint, and marketed features of sport, baby, and body site. Barplot showing the mean number of NAC‐80 allergens from the sunscreen inactive ingredient list for each of the product categories: Green represents comparison of inactive ingredient allergen load by UV filter (organic vs. inorganic vs. combination), yellow represents vehicle (lotion, stick, spray), dark blue represents non‐tinted vs. tinted, magenta represents non‐sport vs. sport, brown represents age (adult vs. baby), and light blue represents region (body vs. face). A one‐way ANOVA was used for each three‐level category comparison (UV filter and vehicle), and the rest of the comparisons were performed using pairwise *t*‐tests. Black vertical lines represent the standard error of the mean (SEM). Asterisks denote significance (*p < 0.05, **p < 0.01, ***p < 0.001; Tukey‐adjusted for ANOVA and FDR‐corrected for pairwise *t*‐tests).

We asked whether allergen load of inactive ingredients differed meaningfully across key sunscreen categories. First, we compared allergen burden of inactive ingredients by UV filter—organic, inorganic and combination. Organic sunscreens had the highest mean allergen count of inactive ingredients (3.12), followed by combination (3.00) and inorganic (1.45) (see Table S2 for mean inactive ingredient allergens by category). This difference was significant (*F*(2, 173) = 34.19, *p* < 0.001), with post hoc tests confirming higher inactive ingredient allergen counts in organic vs. inorganic products (*p* < 0.001) and combination vs. inorganic products (*p* < 0.001). There was no difference between organic and combination (*p* = 0.94). Of note, combination sunscreens (sd = 1.84) had a much higher standard deviation compared to organic (1.35) and inorganic (0.99) products (Figure 3, green bars). The most common allergen within each category is shown in Table S3.

We then compared vehicles. Stick sunscreens had the fewest inactive ingredient allergens (1.29), followed by lotion (2.57) and spray (2.76). A one‐way ANOVA found a significant effect of vehicle type (F(2,173) = 5.49, *p* = 0.005). Stick formulations had significantly fewer allergens than both spray (*p* = 0.005) and lotion (*p* = 0.006), with no difference between spray and lotion (*p* = 0.8) (Figure 3, yellow bars).

In pairwise *t*‐tests of marketing label‐based categories, tinted sunscreens had significantly fewer inactive ingredient allergens than non‐tinted (1.99 vs. 2.85, *p* < 0.001, FDR‐adjusted). Sport‐labelled products had significantly more inactive ingredient allergens than non‐sport (3.03 vs. 2.38, *p* = 0.023, FDR‐adjusted), and face products had significantly fewer inactive ingredient allergens than body products (2.2 vs. 2.71, *p* = 0.044, FDR‐adjusted). The only category that we did not observe significant differences in number of allergens in inactive ingredients was sunscreens marketed for baby versus adult (1.86 vs. 2.56, *p* = 0.061, FDR‐adjusted), although baby trended lower (Figure 3, dark blue, magenta, brown, light blue bars).

**FIGURE 4:**
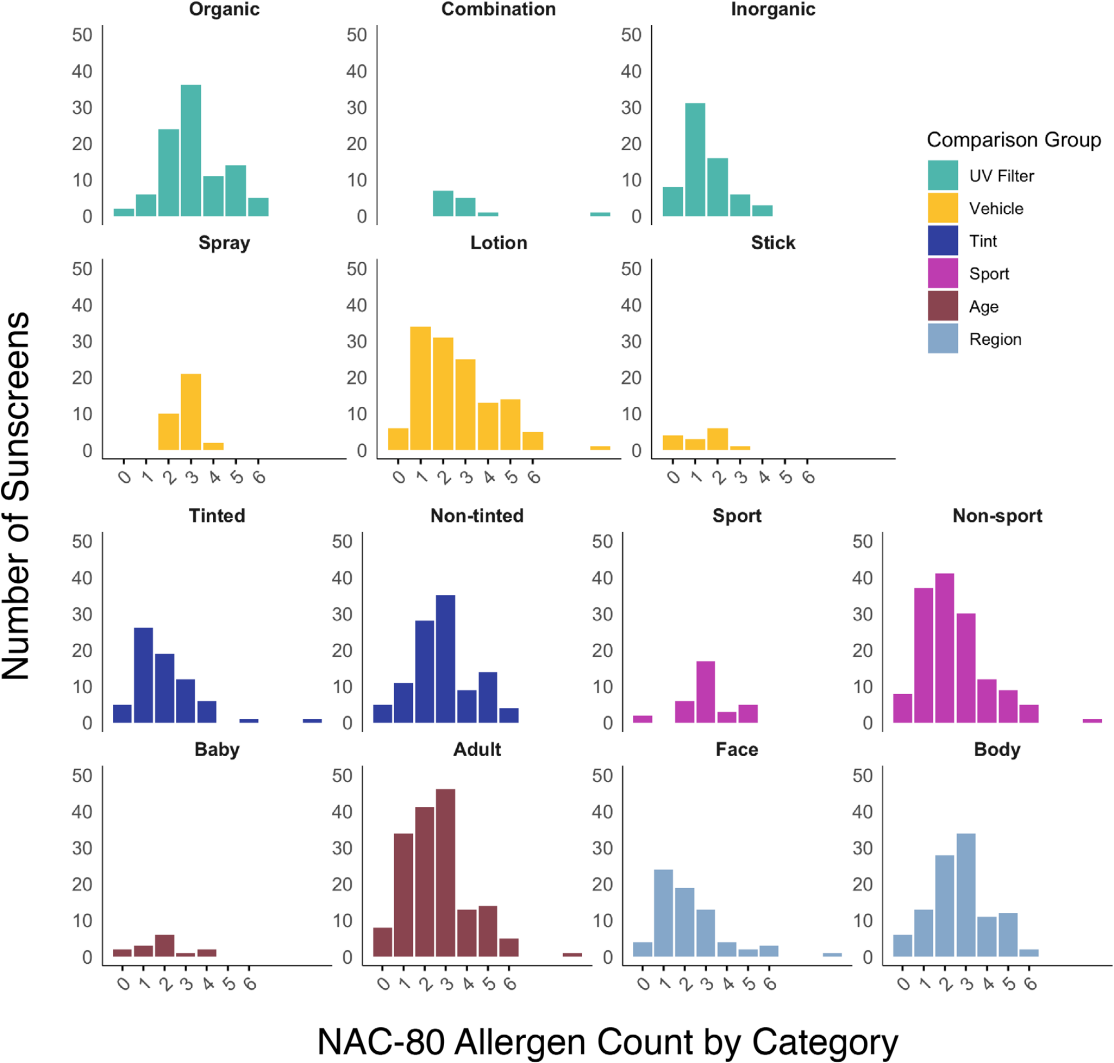
Distribution of NAC‐80 allergens (from inactive ingredient lists) by sunscreen product category. Mini histograms display the count of sunscreens containing 0–9 NAC‐80 allergens, stratified by category. Spray sunscreens showed tightly clustered distributions (97% had 2 or 3 allergens), while inorganic and tinted sunscreens skewed toward lower allergen counts. In contrast, organic, face, and lotion sunscreens showed broader distributions. Categories are colour‐coded to match the groupings used throughout the manuscript.

Finally, histogram plots of inactive ingredient allergen distribution within product categorizations (Figure 4) showed that some categories had more consistent allergen counts than others. Spray sunscreens revealed a tightly clustered distribution, with 97% containing two or three allergens (range: 2–4). In contrast, stick, lotion and non‐tinted products displayed broader variation. Inorganic sunscreens skewed toward lower allergen counts, while organic sunscreens were more evenly distributed from one to five allergens. In most categories, distribution shape mirrored the mean allergen count: lower means reflected left‐skewed, narrower distributions (e.g., tinted vs. non‐tinted histograms).

## 4 Discussion

Only 6% of all best‐selling sunscreens in the U.S. were free of NAC‐80 allergens in their inactive ingredients. We found significant variation in allergen burden of inactive ingredients across sunscreen categories. Organic, sport and spray formulations contained the most allergens; inorganic, baby and stick formulations the least.

Prior studies have evaluated allergen content in sunscreen formulations, including a recent analysis by Divyalakshmi et al. [7] examining commonly accessed sunscreens in India. Similar to our findings, they observed that inorganic (mineral) sunscreens contained fewer allergens than organic (chemical) or combination formulations. However, their analysis included both active and inactive ingredients and screened a broader set of allergens, whereas our study focused specifically on inactive ingredients using the NAC‐80 panel, which is directly relevant to patch testing in North America. In addition, our analysis extends prior work by examining allergen burden across clinically relevant product subtypes, such as vehicle (spray vs. stick) and marketed use (baby, sport, tinted), allowing for more targeted counselling recommendations.

The most common allergen in our dataset was vitamin E, found in 83% of sunscreens (146/176) (as tocopherol and/or tocopheryl acetate). Vitamin E functions as an antioxidant or skin‐conditioning agent and is commonly found in cosmetic products. Acrylate‐containing copolymers and crosspolymers, which are often used as thickening or stabilizing agents to enhance the shelf life of a product, were found in 79% (139/176) of products, and fragrance in 44% (77/176).

Fragrance is often included in products to increase cosmetic appeal, despite their high sensitization rates in patch testing: The NACDG patch test results from 2021 to 2022 reported positive ACD reactions to fragrance mix I in 7.9% of patients and fragrance mix II in 3.0% [8]. If we include agents that are often used as fragrance without being labelled as such, including benzyl alcohol, limonene and linalool, the number of sunscreens in our dataset with fragrance or fragrance‐adjacent allergens rises to 58% (102 products). This suggests fragrance exposure might be underestimated when only the word ‘fragrance’ is counted, and that consumers should be aware that a product labelled as ‘fragrance free’ might still contain functional fragrance allergens known to cause ACD.

The FDA provides guidance on how to choose and apply sunscreen and even includes a section on ingredients, but only lists the common active ingredients (UV filters), even though they note that sunscreens contain both active and inactive ingredients. Given that many allergens exist within the inactive component, listing them is important.

Our findings suggest that the vehicle (e.g., spray vs. stick) and the intended use population (e.g., baby, sport) correlate with allergen load, which may be useful when counselling patients with sensitive skin. For example, baby, inorganic and stick sunscreens all contained the least number of allergens, while organic, spray and sport sunscreens had the highest allergen burden. In practice, these findings may help dermatologists recommend sunscreens with lower inactive ingredient allergen burden, such as inorganic, tinted or stick formulations, for patients with a history of ACD or sensitive skin.

Our findings also speak to a broader tension between tolerability and usability. While sprays and organic sunscreens were among the most allergenic in our analysis, they are also among the most popular. Spray formulations in particular are favoured by consumers for being less greasy and easier to apply [9, 10]. Similarly, organic sunscreens are more cosmetically elegant and may encourage adherence [11, 12]. Balancing short‐term adherence (which product patients are willing to use) with long‐term tolerability (which product is less likely to cause sensitization) is an important clinical consideration.

From a counselling standpoint, clinicians should consider discussing with patients that sunscreens contain both active and inactive components and tailor recommendations to include both patient preference and skin sensitivity. For example, for patients with a history of ACD or atopic dermatitis, steering toward less allergenic formulations such as inorganic or tinted sunscreens, and avoiding organic or ‘sports’ labelling. More broadly, this tension between tolerability and usability highlights the need for patient education around the full ingredient profile of sunscreens, not just actives.

Underlying these choices is a regulatory gap. Only two UVA filters, zinc oxide and avobenzone, are US FDA monographed filters. The FDA currently lists 16 approved UV filters (2 Generally Recognized as Safe and Effective [GRASE], 2 not GRASE and 12 with insufficient safety data), whereas the European Commission permits 29 [13]. The difference stems from regulations: in the U.S., sunscreens are regulated as over‐the‐counter drugs, while in Europe they are considered cosmetics. This restricts the availability of newer UV filters that are photostable and broad spectrum in the U.S. market [14], which may in turn increase reliance on inactive ingredients to improve user experience.

This study has limitations. We focused on best‐selling sunscreens from three major U.S. retailers in May 2024. As a result, niche brands and international products were not included, which may limit generalizability beyond the U.S. retail market. Ingredient lists may not reflect current formulations, and not all products listed complete ingredients online. Because sunscreen formulations and retailer inventories change over time, reproducibility is inherently limited, and repeated analyses may help track trends in inactive ingredient allergens. While NAC‐80 allergens are specific, we allowed broader matching (e.g., acrylates), which may overestimate true allergen burden.

To support informed decision‐making, we developed www.NAC80.com, a free web tool that allows users to input sunscreen ingredients and flag any NAC‐80 allergens present (see Supporting Information for details and example use). Our goal is to provide both clinicians and patients with a practical, accessible way to identify lower‐risk products.

## 5 Conclusion

Allergens are common in best‐selling sunscreens, including in inactive ingredients and vary substantially across product categories. Dermatologists should consider allergen profiles of all listed ingredients when recommending sunscreens, especially for patients with a history of ACD or sensitive skin. Steering patients towards lower‐risk formulations, such as inorganic, baby, or stick sunscreens, may improve tolerability and long‐term adherence without compromising UV protection [15]. Finally, a free web tool (www.NAC80.com) is available to help users check sunscreen inactive ingredients for NAC‐80 allergens.

## Funding

The authors have nothing to report.

## Ethics Statement

This study did not involve human participants, identifiable human data, or animals, and thus did not require institutional review board (IRB) approval. All data used were publicly available or deidentified, and no patient consent was required. This work has not been previously published and is not under consideration for publication elsewhere.

## Conflicts of Interest

The authors declare no conflicts of interest.

## Supporting information


**Data S1:** cod70141‐sup‐0001‐Supinfo.docx.

**Figure S1:** Top 19 most common NAC‐80 allergens found in **inactive ingredients** of best‐selling sunscreens, grouped by chemical class for clarity. Categories such as ‘Vitamin E’ include both tocopherol and tocopheryl acetate; ‘Acrylates’ includes all acrylate‐ and methacrylate‐containing compounds. Parabens include all paraben‐containing compounds.

**Table S1:** cod70141‐sup‐0003‐TableS1.docx.

**Table S2:** cod70141‐sup‐0004‐TableS2.docx.

**Table S3:** Top allergen by category

**Table S4:** Unique NAC‐80 allergens identified in inactive ingredients (*n* = 49)

## Data Availability

The data that support the findings of this study are available from the corresponding author upon reasonable request.
