# Supplementary material for: Allergen Content of Inactive Ingredients in Best‐Selling Sunscreens: A Comparison of Key Product Features
Source: Contact Dermatitis. 2026 Apr 12;95(2):200–6. doi: 10.1111/cod.70141 (PMC13327199; doi:10.1111/cod.70141)
Supplement: Supplementary file 1 — Data S1: cod70141‐sup‐0001‐Supinfo.docx. [file COD-95-200-s001.docx]

**Supplemental methods**

For consistency with clinical patch testing standards, individual parabens (e.g., methylparaben, ethylparaben, propylparaben) were grouped and counted as one allergen (“Paraben mix”), based on their overlapping sensitization profiles and classification in the NAC-80 panel.^1,2^ In contrast, individual acrylates (e.g., ethyl acrylate, hydroxyethyl acrylate, methyl methacrylate) were counted separately to align with patch testing guidelines that treat these compounds as distinct allergens due to their variable cross-reactivity and incomplete copositivity.^3,4^ Tocopherol and tocopheryl acetate were counted as separate allergens due to incomplete cross-reactivity and reported cases of isolated sensitization to each compound.^5^ We counted linalool and limonene as individual allergens, consistent with their inclusion in the NAC-80 series, despite also being classified as fragrance components.

To simplify interpretation for visualization, we grouped allergens with similar chemical structures or naming conventions into broader categories (Figure 2, main results). This grouping was used solely for summary display; all statistical analyses were performed using the original, uncategorized allergen names. Specifically, acrylate- and methacrylate-containing compounds were grouped as “Acrylates,” parabens (e.g., methylparaben, propylparaben) as “Parabens,” tocopherol and tocopheryl acetate as “Vitamin E (Tocopherol),” and fragrance-related entries as “Fragrance.” Note that parabens were counted only as 1 allergen even if multiple appeared in 1 product (e.g., if a product contained methylparaben and propylparaben, this was still only counted as 1 allergen due to sensitivity profile overlap discussed in paragraph above.

Regarding comparing UV filters, sunscreens were categorized as “organic” (i.e., chemical) if their active ingredients contained octocrylene, avobenzone, octisalate, homosalate, oxybenzone, octinoxate, ensulizole, Uvinul A Plus, Uvinul T 150, Enzacamene, Tinosorb S, Tinosorb M, Mexoryl XL, Mexoryl SX, and/or Parsol SLX. Sunscreens were categorized as “inorganic” (i.e., physical, or mineral) if their active ingredients only contained zinc oxide and/or titanium dioxide. “Combination” sunscreens included both organic and inorganic UV filters. Of note, this list includes products with both FDA-approved and non–FDA-approved filters as our focus was on ingredient content in best-selling products available to consumers, regardless of regulatory status.

**Text-matching algorithm**

Inactive ingredient lists were processed using an in-house text-matching algorithm implemented in R. Ingredient strings were split on commas, trimmed, converted to lowercase, and stripped of parenthetical text prior to exact string matching against a predefined NAC-80 reference list from the Chemotechnique Diagnostics NAC-80 panel (North American 80 Comprehensive Series).

In addition to exact matching, rule-based pattern searches were used to flag acrylate-containing ingredients (substring “acrylate”), fragrance-related terms (“fragrance,” “parfum,” and common misspellings), and selected adjacent ingredients (e.g., tocopheryl acetate; limonene/linalool without hydroperoxides; and mix-related terms including paraben/parabens, mercapto, carba, compositae, etc.). To minimize the risk of missed allergens, ingredient lists were manually cross-referenced with the NAC-80 reference list to ensure appropriate classification. The full text-matching algorithm, NAC-80 reference list, and example input/output are available at <https://github.com/emilyjlevin/SunscreenAllergenCode>.


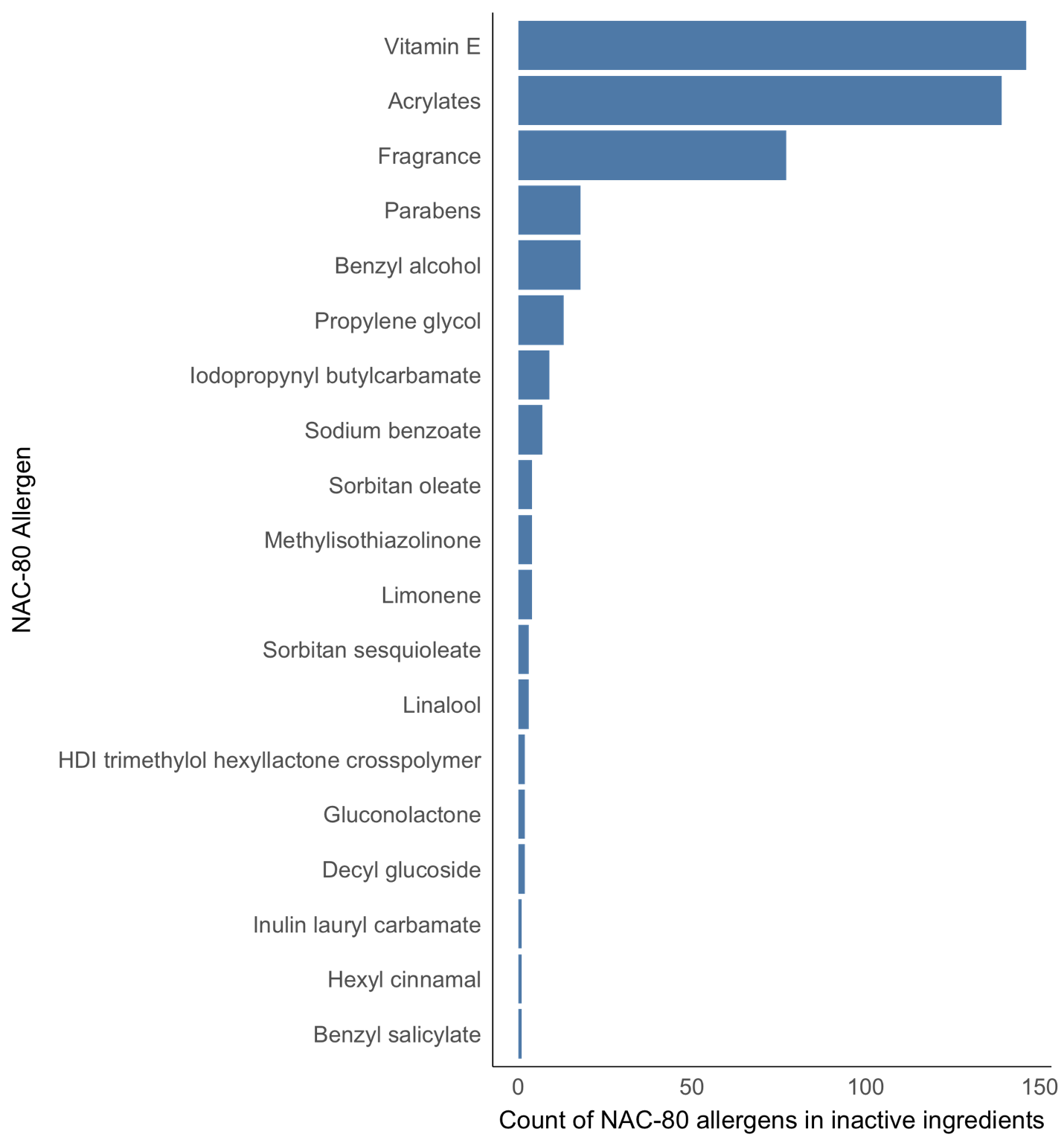


**Supplementary** **Figure 1** Top 19 most common NAC-80 allergens found in **inactive ingredients** of best-selling sunscreens, grouped by chemical class for clarity. Categories such as “Vitamin E” include both tocopherol and tocopheryl acetate; “Acrylates” includes all acrylate- and methacrylate-containing compounds. Parabens include all paraben-containing compounds.

**Allergen category breakdown**

To look at frequency of allergens, we created a histogram (Supplementary Figure 1). In order to simplify interpretation, we grouped allergens with similar chemical structures or naming conventions into broader categories for visualization (Figure 2, main results). This grouping was used solely for summary display; all statistical analyses were performed using the original, uncategorized allergen names. Specifically, acrylate- and methacrylate-containing compounds were grouped as “Acrylates,” parabens (e.g., methylparaben, propylparaben) as “Parabens,” tocopherol and tocopheryl acetate as “Vitamin E (Tocopherol),” and fragrance-related entries as “Fragrance.”

**Active ingredient tabulation**We manually reviewed the active ingredient list for all sunscreens included in our dataset (N = 176). Zinc oxide and titanium dioxide were categorized as inorganic filters, while the following agents were categorized as organic filters. The full count of each ingredient is tabulated below.

**Supplementary Table 1.**

| **Active ingredient (generic name)** | **INCI name** | **Count** |
| --- | --- | --- |
| Octisalate | Ethylhexyl salicylate | 91 |
| Octocrylene | Octocrylene | 91 |
| Avobenzone | Butyl methoxydibenzoylmethane | 90 |
| Homosalate | Homosalate | 85 |
| Zinc oxide | Zinc oxide | 72 |
| Titanium dioxide | Titanium dioxide | 40 |
| Octinoxate | Ethylhexyl methoxycinnamate | 20 |
| Oxybenzone | Benzophenone-3 | 12 |
| Uvinul A Plus | Diethylamino hydroxybenzoyl hexyl benzoate | 4 |
| Ensulizole | Phenylbenzimidazole sulfonic acid | 3 |
| Uvinul T 150 | Ethylhexyl triazone | 3 |
| Tinosorb S | Bis-ethylhexyloxyphenol methoxyphenyl triazine | 3 |
| Tinosorb M | Methylene bis-benzotriazolyl tetramethylbutylphenol | 1 |
| Enzacamene | 4-methylbenzylidene camphor | 1 |
| Parsol SLX | Polysilicone-15 | 1 |
| Mexoryl XL | Drometrizole trisiloxane | 1 |
| Mexoryl SX | Terephthalylidene dicamphor sulfonic acid | 1 |

**Supplementary Table 2.**

| **Category** | **Central Tendency (Mean ± SD)** |
| --- | --- |
| Organic | 3.12 ± 1.35 |
| Sport | 3.03 ± 1.21 |
| Combination | 3.00 ± 1.84 |
| Non-tinted | 2.85 ± 1.43 |
| Spray | 2.76 ± 0.56 |
| Body | 2.71 ± 1.39 |
| Lotion | 2.57 ± 1.65 |
| Adult | 2.56 ± 1.51 |
| Non-sport | 2.38 ± 1.54 |
| Face | 2.20 ± 1.62 |
| Tinted | 1.99 ± 1.46 |
| Baby | 1.86 ± 1.23 |
| Inorganic | 1.45 ± 0.99 |
| Stick | 1.29 ± 0.99 |

**Supplementary table 3. Top allergen by category**

| **Category** | **Top allergen** |
| --- | --- |
| Organic | Fragrance |
| Combination | Tocopheryl acetate |
| Inorganic | Tocopherol |
| Spray | Fragrance |
| Lotion | Tocopherol/tocopheryl acetate (tie) |
| Stick | Fragrance/Tocopherol (tie) |
| Tinted | Tocopherol |
| Non-tinted | Fragrance |
| Sport | Fragrance |
| Non-sport | Tocopherol |
| Baby | Fragrance |
| Adult | Fragrance |
| Face | Tocopherol/Tocopheryl acetate (tie) |
| Body | Fragrance |

**Example Usage of NAC80.com**
Users can copy and paste a comma-separated list of ingredients (e.g., water, zinc oxide, butyloctyl salicylate, tocopherol) into the “Check My Ingredients” tool. The tool flags any NAC-80 allergens, fragrance components, and potentially adjacent ingredients (e.g., tocopheryl acetate, limonene, linalool) and returns a list of matches beneath the input box.

**Supplementary Table 4. Unique NAC-80 allergens identified in inactive ingredients (n = 49)**

| **Allergen** | **Sunscreen products (n)** |
| --- | --- |
| fragrance | 77 |
| tocopherol | 74 |
| tocopheryl_acetate | 72 |
| styrene-acrylates_copolymer | 22 |
| acrylates-octylacrylamide_copolymer | 19 |
| benzyl_alcohol | 18 |
| sodium_polyacrylate | 15 |
| acrylates-c10-30_alkyl_acrylate_crosspolymer | 14 |
| acrylates-c12-22_alkyl_methacrylate_copolymer | 13 |
| propylene_glycol | 13 |
| hydroxyethyl_acrylate-sodium_acryloyldimethyl_taurate_copolymer | 12 |
| va-butyl_maleate-isobornyl_acrylate_copolymer | 10 |
| acrylates-dimethicone_copolymer | 9 |
| iodopropynyl_butylcarbamate | 9 |
| sodium_benzoate | 7 |
| methylparaben | 6 |
| propylparaben | 6 |
| ethylparaben | 4 |
| limonene | 4 |
| methylisothiazolinone | 4 |
| sorbitan_oleate | 4 |
| linalool | 3 |
| polyacrylate-13 | 3 |
| poly_c10-30_alkyl_acrylate | 3 |
| sorbitan_sesquioleate | 3 |
| c12-22_alkyl_acrylate-hydroxyethylacrylate_copolymer | 2 |
| decyl_glucoside | 2 |
| gluconolactone | 2 |
| hdi-trimethylol_hexyllactone_crosspolymer | 2 |
| methyl_methacrylate_crosspolymer | 2 |
| polymethyl_methacrylate | 2 |
| acrylate-octylacrylamide_copolymer | 1 |
| acrylates-c10-30_alkyl_acrylate_crosspolymer_glyceryl_behenate | 1 |
| acrylates_copolymer | 1 |
| acrylates-polytrimethylsiloxymethacrylate_copolymer | 1 |
| benzyl_salicylate | 1 |
| butylparaben | 1 |
| ethylene-methacrylate_copolymer | 1 |
| hexyl_cinnamal | 1 |
| hydroxyethyl_acrylate | 1 |
| inulin_lauryl_carbamate | 1 |
| isobutylparaben | 1 |
| lauryl_methacrylate-sodium_methacrylate_crosspolymer | 1 |
| methyl_methacrylate-glycol_dimethacrylate_crosspolymer | 1 |
| polyacrylate_cross-_polymer-6 | 1 |
| sodium_acrylates_crosspolymer-2 | 1 |
| sodium_acrylate-sodium_acryloyldimethyl_taurate_copolymer | 1 |
| styrene-acrylates | 1 |
| vp-acrylates-lauryl_methacrylate_copolymer | 1 |

**References**

1. Fonacier, L. *et al.* Contact Dermatitis: A Practice Parameter-Update 2015. *J. Allergy Clin. Immunol. Pract.* **3**, S1–S39 (2015).

2. Yang, Y. W. *et al.* Systematic Identification of Copositivity Groups in Standard Series Patch Testing Through Hierarchical Clustering. *JAMA Dermatology* **159**, 945–952 (2023).

3. Aalto-Korte, K., Pesonen, M., Kuuliala, O., Alanko, K. & Jolanki, R. Contact allergy to aliphatic polyisocyanates based on hexamethylene-1,6- diisocyanate (HDI). *Contact Dermatitis* **63**, 357–363 (2010).

4. Spencer, A., Gazzani, P. & Thompson, D. A. Acrylate and methacrylate contact allergy and allergic contact disease: a 13-year review. *Contact Dermatitis* **75**, 157–164 (2016).

5. de Groot, A. C. *et al.* Allergic contact dermatitis from tocopheryl acetate in cosmetic creams. *Contact Dermatitis* **25**, 302–304 (1991).
