# Supplementary figures and images for: Allergen Content of Inactive Ingredients in Best‐Selling Sunscreens: A Comparison of Key Product Features
Source: Contact Dermatitis. 2026 Apr 12;95(2):200–6. doi: 10.1111/cod.70141 (PMC13327199; doi:10.1111/cod.70141)

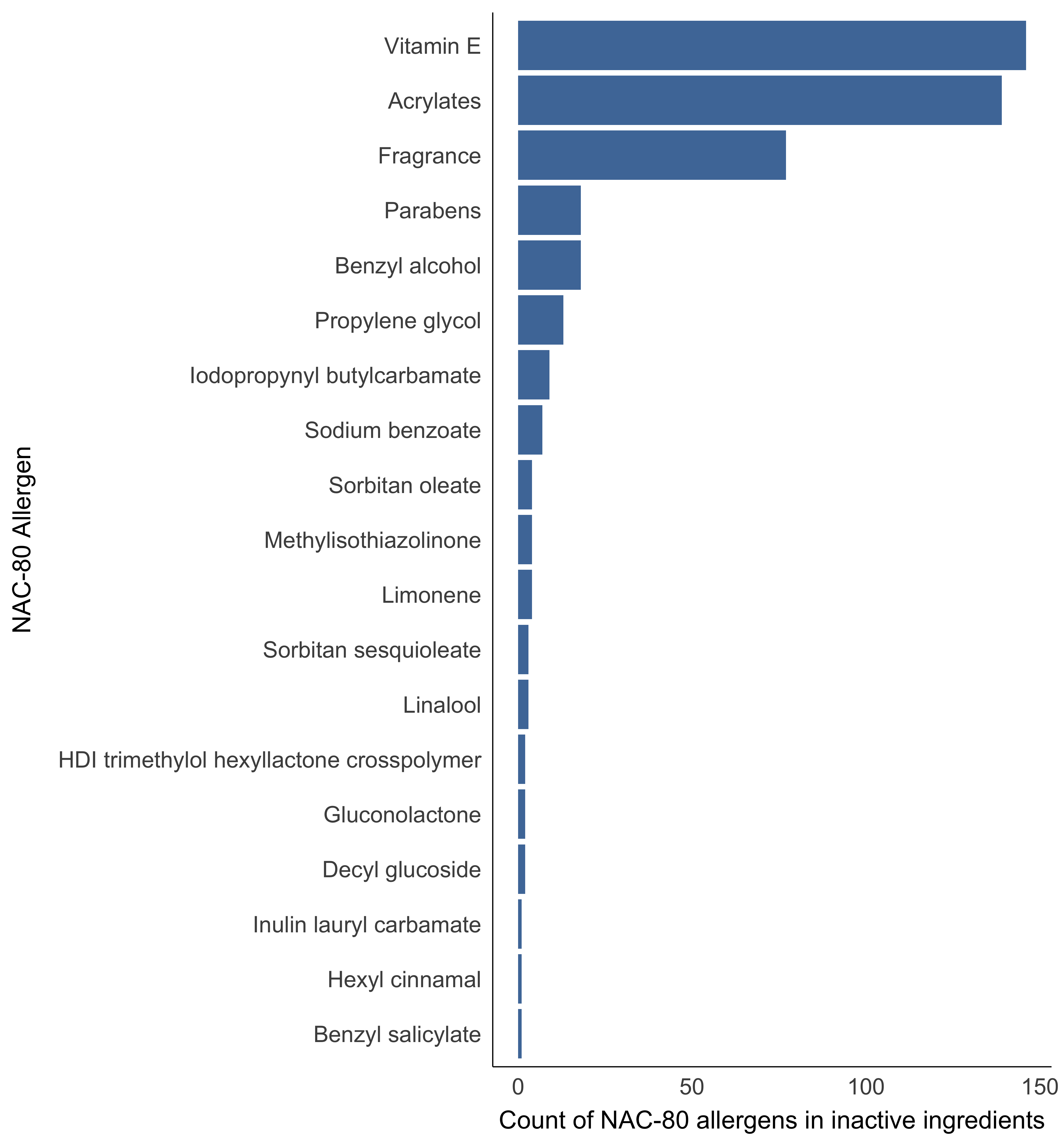

Supplement: Supplementary file 2 — Figure S1: Top 19 most common NAC‐80 allergens found in inactive ingredients of best‐selling sunscreens, grouped by chemical class for clarity. Categories such as ‘Vitamin E’ include both tocopherol and tocopheryl acetate; ‘Acrylates’ includes all acrylate‐ and methacrylate‐containing compounds. Parabens include all paraben‐containing compounds. [file COD-95-200-s004.png]
