# Supplementary material for: Allergen Content of Inactive Ingredients in Best‐Selling Sunscreens: A Comparison of Key Product Features
Source: Contact Dermatitis. 2026 Apr 12;95(2):200–6. doi: 10.1111/cod.70141 (PMC13327199; doi:10.1111/cod.70141)
Supplement: Supplementary file 3 — Table S1: cod70141‐sup‐0003‐TableS1.docx. [file COD-95-200-s006.docx]

**Supplementary Table 1.**

| **Active ingredient (generic name)** | **INCI name** | **Count** |
| --- | --- | --- |
| Octisalate | Ethylhexyl salicylate | 91 |
| Octocrylene | Octocrylene | 91 |
| Avobenzone | Butyl methoxydibenzoylmethane | 90 |
| Homosalate | Homosalate | 85 |
| Zinc oxide | Zinc oxide | 72 |
| Titanium dioxide | Titanium dioxide | 40 |
| Octinoxate | Ethylhexyl methoxycinnamate | 20 |
| Oxybenzone | Benzophenone-3 | 12 |
| Uvinul A Plus | Diethylamino hydroxybenzoyl hexyl benzoate | 4 |
| Ensulizole | Phenylbenzimidazole sulfonic acid | 3 |
| Uvinul T 150 | Ethylhexyl triazone | 3 |
| Tinosorb S | Bis-ethylhexyloxyphenol methoxyphenyl triazine | 3 |
| Tinosorb M | Methylene bis-benzotriazolyl tetramethylbutylphenol | 1 |
| Enzacamene | 4-methylbenzylidene camphor | 1 |
| Parsol SLX | Polysilicone-15 | 1 |
| Mexoryl XL | Drometrizole trisiloxane | 1 |
| Mexoryl SX | Terephthalylidene dicamphor sulfonic acid | 1 |
