# Supplementary material for: Allergen Content of Inactive Ingredients in Best‐Selling Sunscreens: A Comparison of Key Product Features
Source: Contact Dermatitis. 2026 Apr 12;95(2):200–6. doi: 10.1111/cod.70141 (PMC13327199; doi:10.1111/cod.70141)
Supplement: Supplementary file 4 — Table S2: cod70141‐sup‐0004‐TableS2.docx. [file COD-95-200-s005.docx]

**Supplementary Table 2.**

| **Category** | **Central Tendency (Mean ± SD)** |
| --- | --- |
| Organic | 3.12 ± 1.35 |
| Sport | 3.03 ± 1.21 |
| Combination | 3.00 ± 1.84 |
| Non-tinted | 2.85 ± 1.43 |
| Spray | 2.76 ± 0.56 |
| Body | 2.71 ± 1.39 |
| Lotion | 2.57 ± 1.65 |
| Adult | 2.56 ± 1.51 |
| Non-sport | 2.38 ± 1.54 |
| Face | 2.20 ± 1.62 |
| Tinted | 1.99 ± 1.46 |
| Baby | 1.86 ± 1.23 |
| Inorganic | 1.45 ± 0.99 |
| Stick | 1.29 ± 0.99 |
