# Supplementary material for: Allergen Content of Inactive Ingredients in Best‐Selling Sunscreens: A Comparison of Key Product Features
Source: Contact Dermatitis. 2026 Apr 12;95(2):200–6. doi: 10.1111/cod.70141 (PMC13327199; doi:10.1111/cod.70141)
Supplement: Supplementary file 5 — Table S3: Top allergen by category [file COD-95-200-s002.docx]

**Supplementary table 3. Top allergen by category**

| **Category** | **Top allergen** |
| --- | --- |
| Organic | Fragrance |
| Combination | Tocopheryl acetate |
| Inorganic | Tocopherol |
| Spray | Fragrance |
| Lotion | Tocopherol/tocopheryl acetate (tie) |
| Stick | Fragrance/Tocopherol (tie) |
| Tinted | Tocopherol |
| Non-tinted | Fragrance |
| Sport | Fragrance |
| Non-sport | Tocopherol |
| Baby | Fragrance |
| Adult | Fragrance |
| Face | Tocopherol/Tocopheryl acetate (tie) |
| Body | Fragrance |
