# Supplementary material for: Allergen Content of Inactive Ingredients in Best‐Selling Sunscreens: A Comparison of Key Product Features
Source: Contact Dermatitis. 2026 Apr 12;95(2):200–6. doi: 10.1111/cod.70141 (PMC13327199; doi:10.1111/cod.70141)
Supplement: Supplementary file 6 — Table S4: Unique NAC‐80 allergens identified in inactive ingredients (n = 49) [file COD-95-200-s003.docx]

**Supplementary Table 4. Unique NAC-80 allergens identified in inactive ingredients (n = 49)**

| **Allergen** | **Sunscreen products (n)** |
| --- | --- |
| fragrance | 77 |
| tocopherol | 74 |
| tocopheryl_acetate | 72 |
| styrene-acrylates_copolymer | 22 |
| acrylates-octylacrylamide_copolymer | 19 |
| benzyl_alcohol | 18 |
| sodium_polyacrylate | 15 |
| acrylates-c10-30_alkyl_acrylate_crosspolymer | 14 |
| acrylates-c12-22_alkyl_methacrylate_copolymer | 13 |
| propylene_glycol | 13 |
| hydroxyethyl_acrylate-sodium_acryloyldimethyl_taurate_copolymer | 12 |
| va-butyl_maleate-isobornyl_acrylate_copolymer | 10 |
| acrylates-dimethicone_copolymer | 9 |
| iodopropynyl_butylcarbamate | 9 |
| sodium_benzoate | 7 |
| methylparaben | 6 |
| propylparaben | 6 |
| ethylparaben | 4 |
| limonene | 4 |
| methylisothiazolinone | 4 |
| sorbitan_oleate | 4 |
| linalool | 3 |
| polyacrylate-13 | 3 |
| poly_c10-30_alkyl_acrylate | 3 |
| sorbitan_sesquioleate | 3 |
| c12-22_alkyl_acrylate-hydroxyethylacrylate_copolymer | 2 |
| decyl_glucoside | 2 |
| gluconolactone | 2 |
| hdi-trimethylol_hexyllactone_crosspolymer | 2 |
| methyl_methacrylate_crosspolymer | 2 |
| polymethyl_methacrylate | 2 |
| acrylate-octylacrylamide_copolymer | 1 |
| acrylates-c10-30_alkyl_acrylate_crosspolymer_glyceryl_behenate | 1 |
| acrylates_copolymer | 1 |
| acrylates-polytrimethylsiloxymethacrylate_copolymer | 1 |
| benzyl_salicylate | 1 |
| butylparaben | 1 |
| ethylene-methacrylate_copolymer | 1 |
| hexyl_cinnamal | 1 |
| hydroxyethyl_acrylate | 1 |
| inulin_lauryl_carbamate | 1 |
| isobutylparaben | 1 |
| lauryl_methacrylate-sodium_methacrylate_crosspolymer | 1 |
| methyl_methacrylate-glycol_dimethacrylate_crosspolymer | 1 |
| polyacrylate_cross-_polymer-6 | 1 |
| sodium_acrylates_crosspolymer-2 | 1 |
| sodium_acrylate-sodium_acryloyldimethyl_taurate_copolymer | 1 |
| styrene-acrylates | 1 |
| vp-acrylates-lauryl_methacrylate_copolymer | 1 |
